# Additive Effect of Parathyroid Hormone and Zoledronate Acid on Prevention Particle Wears-Induced Implant Loosening by Promoting Periprosthetic Bone Architecture and Strength in an Ovariectomized Rat Model

**DOI:** 10.3389/fendo.2022.871380

**Published:** 2022-04-25

**Authors:** Chenhe Zhou, Yangxin Wang, Jiahong Meng, Minjun Yao, Huikang Xu, Cong Wang, Fanggang Bi, Hanxiao Zhu, Guang Yang, Mingmin Shi, Shigui Yan, Haobo Wu

**Affiliations:** ^1^ Department of Orthopedic Surgery, The Second Affiliated Hospital, Zhejiang University School of Medicine, Hangzhou, China; ^2^ Orthopedic Research Institute of Zhejiang University, Hangzhou, China; ^3^ Key Laboratory of Motor System Disease Research and Precision Therapy of Zhejiang Province, The Second Affiliated Hospital, Zhejiang University, Hangzhou, China; ^4^ State Key Laboratory for Diagnosis and Treatment of Infectious Diseases, National Clinical Research Center for Infectious Diseases, Collaborative Innovation Center for Diagnosis and Treatment of Infectious Diseases, The First Affiliated Hospital, College of Medicine, Zhejiang University, Hangzhou, China; ^5^ Department of Orthopaedic Surgery, The First Affiliated Hospital of Zhengzhou University, Zhengzhou, China

**Keywords:** implant loosening, osteolysis, osteoporosis, parathyroid hormone (1-34), zoledronate (ZOL)

## Abstract

Implant-generated particle wears are considered as the major cause for the induction of implant loosening, which is more susceptible to patients with osteoporosis. Monotherapy with parathyroid hormone (PTH) or zoledronate acid (ZOL) has been proven efficient for preventing early-stage periprosthetic osteolysis, while the combination therapy with PTH and ZOL has exerted beneficial effects on the treatment of posterior lumbar vertebral fusion and disuse osteopenia. However, PTH and ZOL still have not been licensed for the treatment of implant loosening to date clinically. In this study, we have explored the effect of single or combined administration with PTH and ZOL on implant loosening in a rat model of osteoporosis. After 12 weeks of ovariectomized surgery, a femoral particle-induced periprosthetic osteolysis model was established. Vehicle, PTH (5 days per week), ZOL (100 mg/kg per week), or combination therapy was utilized for another 6 weeks before sacrifice, followed by micro-CT, histology, mechanical testing, and bone turnover examination. PTH monotherapy or combined PTH with ZOL exerted a protective effect on maintaining implant stability by elevating periprosthetic bone mass and inhibiting pseudomembrane formation. Moreover, an additive effect was observed when combining PTH with ZOL, resulting in better fixation strength, higher periprosthetic bone mass, and less pseudomembrane than PTH monotherapy. Taken together, our results suggested that a combination therapy of PTH and ZOL might be a promising approach for the intervention of early-stage implant loosening in patients with osteoporosis.

## Introduction

Arthroplasty has been widely utilized to improve the life quality of patients with end-stage osteoarthritis. However, the occurrence of complications still impacts some patients. Among those complications, implant failure is the most severe one, which might result in revision surgeries eventually. The survival rate of implants is reported to be only 71% at 20 years (1). Aseptic loosening, the major reason for arthroplasty failure, is caused by an inflammatory response to the debris generated from the prosthesis, leading to a promotion of periprosthetic bone loss and pseudomembrane formation, as well as a reduction of prosthesis stability (2, 3). So far, the only treatment for arthroplasty failure is revision surgery, which requires higher technical demand and higher costs but results in a higher complication rate and lower satisfaction rate (4, 5). In the meantime, there is also an essential concern that those patients with revision surgery are likely to be older and frailer, which might result in a higher surgical risk. Therefore, an effective tactic with minimal invasion to prevent prosthetic loosening is of great value.

As reported previously, over 50% of patients undergoing total hip arthroplasty (THA) are combined with osteoporosis (6). Nevertheless, osteoporosis has been proven to negatively regulate implant osseointegration and further increase prosthetic migration (7, 8). Moreover, poor bone quality also increases the prevalence of periprosthetic osteolysis. A study has shown that aseptic loosening is associated with low lumbar bone mineral density (BMD) (9). Additionally, more significant bone loss has been observed in the femoral component in female patients associated with low systemic BMD (10). Excessive osteoclast activities are confirmed to be responsible for both periprosthetic osteolysis and osteoporosis, which are characterized by increasing bone remodeling, especially increasing bone loss (11, 12). Due to the vital role of and similar mechanism with osteoporosis, the utilization of agents with a capacity of regulating bone remodeling has the potential to be an effective approach for implant-loosening prevention.

Many systemic antiosteoporotic agents have been tested to prevent implant loosening by inhibiting periprosthetic osteolysis or improving periprosthetic osseointegration (13). Bisphosphonates, generally regarded as the first-line treatment for osteoporosis, have been verified to demonstrate a beneficial effect on suppressing periprosthetic osteolysis in animal models (14, 15). A clinical study also confirmed that a single infusion of zoledronic acid (ZOL) could reduce the early implant migration in hip arthroplasty (16). Additionally, intermittent parathyroid hormone (PTH) 1-34 is another classical mediation for the treatment of osteoporosis, with the capacity of anabolic effects. Our and other research groups have revealed that intermittent PTH treatment could prevent particle-induced osteolysis *in vivo* (17–19). Recently, several cases reported that PTH has a protective effect on prosthetic loosening clinically (20–22). Therefore, both PTH and ZOL are promising to be therapeutic options for implant loosening. In addition, the combined use of PTH and ZOL has been found to have an additive-promoting effect in several animal models of skeletal diseases, such as posterior lumbar vertebral fusion in ovariectomized (OVX) rats and fracture healing (23, 24). However, to date, PTH and ZOL still have not been licensed to treat implant loosening. Also, bisphosphonate use was found to increase the incidence rate of periprosthetic fractures after THA in patients with normal bone quality (25). As the crucial role of osteoporosis mentioned above in implant loosening, it is reasonable and meaningful to detect the effects of approved antiosteoporotic agents, PTH and ZOL, on early-stage periprosthetic osteolysis in patients with poor bone quality. In this study, we established a particle-induced periprosthetic osteolysis model in OVX rats and further investigated and compared the effects of combined PTH and ZOL with monotherapy on preventing implant loosening.

## Method and Materials

### Preparation of Agents and Materials

PTH (1-34) was obtained from Bachem ( Bubendorf, Switzerland) and dissolved at a concentration of 60 μg/ml with distilled water. ZOL (Sigma–Aldrich, St. Louis, MO, United States) was dissolved to 100 μg/ml with distilled water. Titanium (Ti) rods and the suspension of 30 mg Ti particles (Johnson Matthey, Ward Hill, MA, United States) were gained and prepared as reported in our previous study (26).

### Animal Experiment

All animal care and the entire experiment protocols followed the Guide for the Care and Use of Laboratory Animal published by the United States National Institutes of Health and were approved by the Institutional Animal Care and Use Committee of the Second Affiliated Hospital, Zhejiang University School of Medicine. Female Sprague–Dawley (SD) rats were purchased from Shanghai SLAC Laboratory Animal Co. Ltd, (Shanghai, China) and were maintained in a room with a temperature of 24 ± 2°C, humidity of 60%, and light/dark cycle rhythm of 12 h. Animals were free to access to water and food with a number of two per cage. All rats were assessed every day for visual signs of pain, morbidity, or depression during the whole experiment period. Animals were sacrificed humanely with symptoms above or an acute loss of 10% weight. All efforts we made in this study aimed to minimize the number of animals used and animal suffering.

A total of sixty-six female SD rats weighing 200–250 g were randomly assigned into the sham group (n=13) and OVXS group (n=53). All rats were anesthetized with an intraperitoneal injection of pentobarbital sodium (50 mg/kg). Both the sham surgery and OVX surgery were processed as we reported previously (27). Twelve weeks postoperatively, three rats in each group were selected randomly and sacrificed for radiological and histological analysis to confirm the successful establishment of the osteoporosis model.

The remaining sixty rats were used for the following experiment, including 10 in the sham group and 50 in the OVXS group. Ti rod implantation was performed in the remaining animals. Rats in the sham group were identified as the normal control (NC) group, while rats in the OVXS group were randomly divided into 5 groups (n=10 per group): the OVX group, O+T group, PTH group, ZOL group, and P+Z group. The rat model of implant loosening was established as we reported previously (26). Briefly, Ti rods were implanted into the medullary canal of distal femurs bilaterally after the injection of PBS or 30 mg Ti suspension in the canal. Postoperatively, intra-articular PBS or Ti particle suspension injection was performed at weeks 2 and 4. Ti particles were used in the O+T group, PTH group, ZOL group, and P+Z group, while vehicles were used in the other two groups. One week after surgery, rats were administrated with PTH (60 mg/kg 5 days per week, subcutaneous injection) in the PTH group, or with ZOL (100 μg/kg per week, intraperitoneal injection) in the ZOL group, or with combined PTH (60 mg/kg 5 days per week) and ZOL (100 μg/kg every week) in the P+Z group (combination therapy), and vehicle injection was used in the remaining three groups. After 6 weeks of treatment, rats were euthanasia with an overdose injection of pentobarbital sodium (90 mg/kg), and specimens were collected for further analysis, including biomechanical testing, X-ray examination, micro-CT, histomorphology, and bone turnover analysis. Subcutaneous injections of 10 mg/kg calcein green and 30 mg/kg alizarin red were performed 14 and 4 days before euthanasia, respectively.

### X-Ray Examination and Micro-CT Analysis

Femurs were fixed in 4% (w/v) paraformaldehyde (PFA) for 48 h and used for X-ray examination and micro-CT analysis. X-ray examination was performed using 40 kV and 25 mAs to detect the general radiological manifestations. After X-ray examination, the specimens were subsequently transported for micro-CT scanning. A Scanco μCT100 instrument (Scanco Medical, Bassersdorf, Switzerland) was utilized at 14.8 μm isometric resolution. All parameters of micro-CT scanning and the selection of the region of interest (ROI) were referred to in our previous study (26). The data of bone mineral density (BMD) bone volume/total volume (BV/TV), bone surface/bone volume (BS/BV), connective density (Conn.D), structural model index (SMI), trabecular number (Tb.N), trabecular thickness (Tb.Th), and trabecular separation (Tb.Sp) were collected for quantitative analysis.

### Histomorphological Analysis

Methylmethacrylate-embedded femurs were fixed in 70% alcohol and processed without demineralization as described before (28). Followed by being grounded to 50 μm thick slices, sample blocks were cut into 1 mm thick pieces *via* the cross-section using the IsoMet 5000 instrument (Buehler, Lake Bluff, IL, United States) and attached to plastic slides. Sections located 1 mm below the distal femoral growth plate were recorded under fluorescence microscopy (Leica DM5 500B; Leica Microsystems, Bensheim, Germany), and the parameters for bone formation were calculated with Image J software, including the mineral apposition rate (MAR) and mineral surface/bone surface (MS/BS).

Paraffin-embedded femurs were fixed in 4% PFA for 48 h and decalcified for 2 months in 10% (w/v) ethylene diamine tetraacetic acid (EDTA). Samples collected beyond implant-loosening surgery were cut along the coronal plane, and the others were sliced perpendicularly to the long axis after removing Ti rods. Five-micron-thick sections were cut in the similar region as methylmethacrylate-embedded samples above, followed by being performed with hematoxylin and eosin (H&E) and Masson and tartrate-resistant acid phosphatase (TRAP) staining as previously reported (26). The observation and measurement of samples were performed under light microscopy (Olympus BX51, Tokyo, Japan), and the histological images were evaluated by bone–implant contact (BIC), the ratio of bone area/total area (B.Ar/T.Ar), and mean thickness of the pseudomembrane according to our previous study (26).

Immunohistochemistry (IHC) staining was performed to detect the expression of osteocalcin (OCN) and receptor activator of NF-κB ligand (RANKL) around the Ti rods. The staining was processed according to the suggested protocol of an immunohistochemistry staining accessory kit (Boshide, Wuhan, China).

### Bone Turnover Biomarkers

Before sacrifice, all rats fasted for 12 h. After anesthesia, blood was drawn from the abdominal aorta, and serum was subsequently obtained from the upper layer after blood centrifugation at 425× g for 5 min. Serum type I collagen cross-linked C-terminal telopeptide (CTX-1) and Gla-osteocalcin (GLA-OCN) levels were detected using Ratlaps (CTX-1) enzyme immunoassay (EIA) kit (Immunodiagnostic Systems Limited, Boldon, United Kingdom) and Rat Gla-Osteocalcin High Sensitive EIA Kit (TaKaRa Bio, Otsu, Japan) following the manufacturers’ protocol, respectively.

### Mechanical Testing

Femurs from each group were collected and stored at -20°C, covered with gauze soaked in normal saline. A biomechanical test was performed after specimens were melted completely at 4°C. All samples were prepared, and the subsequent pull-out testing was performed as shown in **Figure 2A** using a Zwick/Roell 2.5 material testing system (Zwick, Ulm, Germany), as we described previously (26). The maximum fixation strength (N) and stiffness (N/m) were recorded and analyzed from the load–displacement curve (29).

### Statistical Analysis

All data were collected and analyzed by two independent researchers blinded to grouping and treatment. ROUT was conducted on the data to identify outliers with GraphPad Prism Version 9.0, and no data points were excluded. Results were shown as mean ± standard deviation (SD) and processed using GraphPad Prism Version 9.0 software. An unpaired t-test was used to analyze the differences between two groups, while one-way ANOVA with *post-hoc* Tukey’s multiple comparison test was used to compare multiple groups. The values (*p < 0.05 and **p < 0.01) were set as the threshold of statistical significance.

## Results

### Confirmation of Ovariectomized-Induced Osteoporosis Model

**Figure 1:**
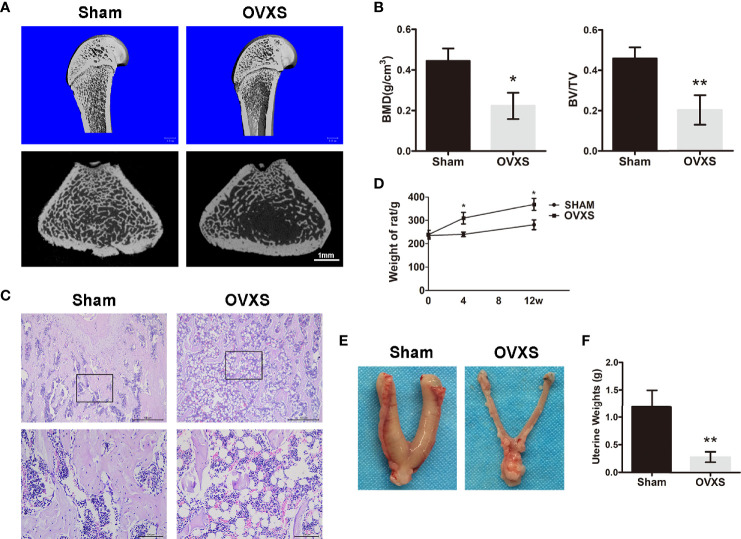
The establishment of the OVX model was confirmed by radiological and histomorphological analysis. **(A)** The representative 3D and 2D micro-CT images of distal femurs were demonstrated from the sham group and the OVXS groups. **(B)** The BMD and BV/TV values of micro-CT images were quantified. **(C)** The representative images of H&E staining from both groups were performed. Upper ×40, lower ×200 magnification. Scale bar = 500 μm (upper) and 100 μm (lower). **(D)** The bodyweight of rats from 0 to 12 weeks after OVX in both groups. The representative images of the uterus **(E)** and weight **(F)** were obtained at the time of sacrifice in both groups. Values are expressed as mean ± SD, n=3; *p<0.05, **p<0.01, compared with the sham group.

To validate whether osteoporosis was induced successfully, three rats from each group (the sham group and OVXS group) were selected randomly for analysis. As shown in **Figures 1A–C** and **Figure S1**, micro-CT and H&E staining data demonstrated a decreased bone mass in the OVXS group compared to the sham group (BMD: t=4.3, df 4, p<0.05; BV/TV: t=4.83, df 4, p<0.01). In addition, bodyweight elevation and uterus atrophy (t=4.964, df 4, p<0.01) further revealed that the OVX-induced osteoporosis model was established successfully (**Figures 1D–F**).

### Preventive Effects of Combined Therapy and Monotherapy on Particle-Induced Implant Fixation

**Figure 2:**
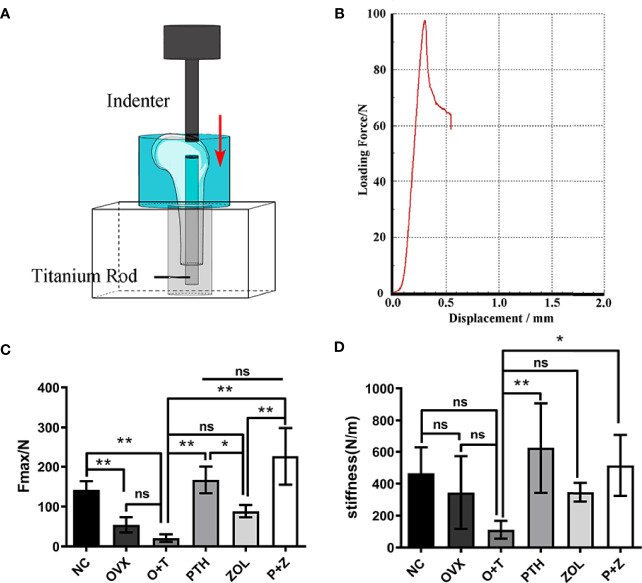
The positive effects of combined or single treatment with PTH and ZOL on inhibiting particle-induced fixation strength loss of the implant in OVX rats. The schematic image of the biomechanical testing device **(A)** and the representative loading force–displacement curve **(B)** were displayed. The maximal fixation strength **(C)** and stiffness **(D)** of samples were collected and analyzed. Values expressed are means ± SD; *p < 0.05, n=5; **p < 0.01, significantly different compared between two groups. "ns", no significant difference.

A biomechanical test was performed to investigate the effect of combination therapy and monotherapy on implant fixation stability. The results were presented as the maximal pull-out force and stiffness. As shown in **Figures 2C, D**, reduced maximal implant pull-out strength was observed in the OVX group compared to the NC group, while no difference was demonstrated in stiffness between the two groups. The lowest maximal implant pull-out strength and stiffness were found in the O+T group compared with the NC and OVX groups. These data indicated that the implant loosening model had been constructed based on the OVX model. After the treatment of PTH alone, or ZOL alone, or combination therapy, significantly improved implant fixation was manifested with increased maximal pull-out force (F=23.4, df 5 and 24, p<0.01) and stiffness (F=4.696, df 5 and 24, p<0.01) in the PTH group and P+Z group, whereas no difference but an increasing trend was found under ZOL alone (**Figures 2C, D**). Moreover, the combination therapy of PTH and ZOL demonstrated a better preventive effect on the maximal pull-out force than the ZOL group. However, no differences have been observed between combination therapy and PTH alone on the maximal pull-out strength. Also, combination therapy has not shown any differences in stiffness among PTH alone and ZOL alone mechanically.

### Effects of Combined Therapy and Monotherapy on Blocking Periprosthetic Destruction of Bone Architecture

**Figure 3:**
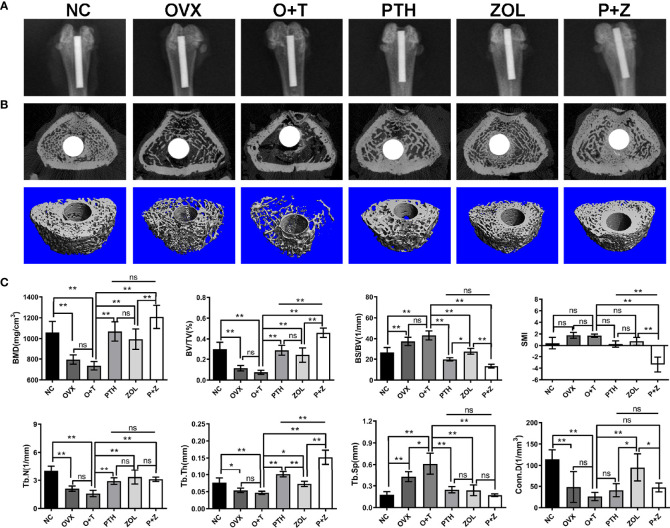
The beneficial effect of combined therapy or monotherapy on preventing particles-induced peri-implant bone loss was observed radiologically in OVX rats. **(A)** The representative X-ray images from the NC, OVX, O+T, PTH, ZOL, and P+Z groups were exhibited. **(B)** The representative 3D and 2D micro-CT images of peri-implant bone mass in distal femurs were demonstrated from the six groups. **(C)** The quantification of the BMD, BV/TV, BS/BV, Conn.D, SMI, Tb.N, Tb.Th and Tb.Sp values was analyzed. Values expressed are means ± SD, n=5; *p < 0.05, **p < 0.01, significantly different compared between two groups. "ns", no significant difference.

Radiological examinations were further utilized to explore the effects of combined therapy and monotherapy on bone architecture. General manifestations were demonstrated using X-ray examination. Implants were appropriately inserted into the medullary canals in **Figure 3A**, and noticeable bone mass loss and radiolucent regions were observed around the implant in the O+T group. After combination or monotherapy of PTH and ZOL, greater peri-implant bone mass in the distal femurs and reduced radiolucent lines were exhibited (**Figure 3A**).

To further confirm the therapeutic effects of combination and monotherapy, distal femurs were analyzed by micro-CT scanning. As revealed in **Figures 3B, C**, significantly decreased peri-implant bone mass was found in the OVX group in comparison with the NC group. Lower peri-implant bone mass in a trend was observed after Ti particle treatment in the OVX rats, compared to rats in the OVX group, according to 3D micro-CT-reconstructed images (**Figure 3B**). Notably, combination therapy and the monotherapy of PTH and ZOL significantly increased peri-implant bone mass, resulting in improved bone architecture (**Figure 3B**). The quantification of micro-CT scanning data was next performed and displayed as BMD, BV/TV, BS/BV, Conn.D, SMI, Tb.N, Tb.Th, and Tb.Sp. As indicated in **Figure 3C**, increased Tb.Sp was observed with the treatment of Ti particles in OVX rats. However, no considerable differences were found between the OVX group and the O+T group in BMD, BV/TV, Tb.N, Tb.Th, and Conn.D (F=10.99, df 5 and 24, p<0.01); a decreased trend could still be observed after the administration of Ti particles. In contrast, with the treatment of combined PTH and ZOL, or monotherapy, remarkable elevations were demonstrated in BMD (F=21.15, df 5 and 24, p<0.01), BV/TV (F=39.03, df 5 and 24, p<0.01), Tb.N (F=18.77, df 5 and 24, p<0.01) and Tb.Th (F=55.72, df 5 and 24, p<0.01), while BS/BV (F=50.71, df 5 and 24, p<0.01) and Tb.Sp (F=24.90, df 5 and 24, p<0.01)values were significantly decreased in comparison with those in the O+T group. Moreover, combined PTH and ZOL therapy exhibited a better preventive effect than PTH alone or ZOL alone on peri-implant bone loss, presented as greater enhancement in BMD, BV/TV, Tb.Th, and reduction in SMI (F=28.87, df 5 and 24, p<0.01). In addition, no differences were observed in most of the parameters in the comparison between the PTH group and the ZOL group, whereas higher BS/BV and Conn.D and lower Tb.Th were shown in the ZOL group (**Figure 3C**).

**Figure 4:**
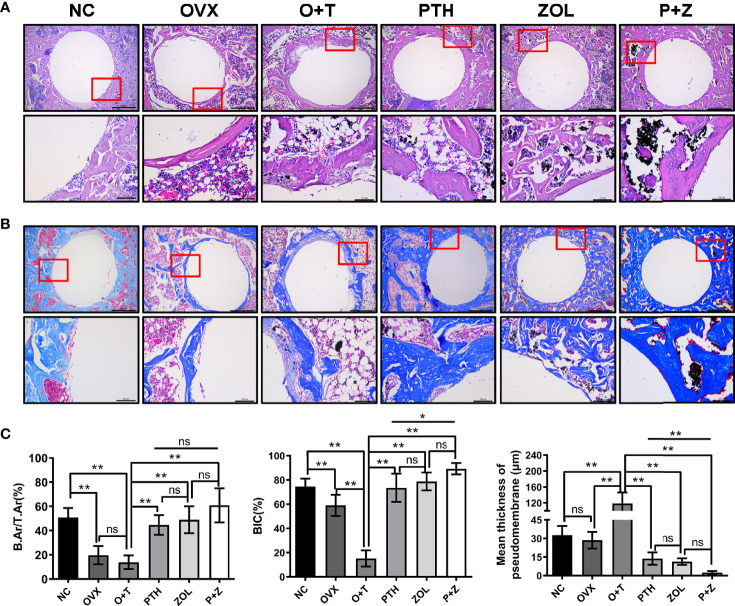
Histomorphology of combined therapy or monotherapy on protecting particle-induced peri-implant osteolysis. **(A)** H&E staining (upper ×40, lower ×200 magnification) and **(B)** Masson staining (upper 40×, lower ×200 magnification) were used for the histological analysis. Scale bar= 500 μm (upper) and 100 μm (lower). **(C)** BIC, B.Ar/T.Ar, and mean thickness of the pseudomembrane were quantified. Values expressed are means ± SD, n=5; **p < 0.01, significantly different compared between two groups. "ns", no significant difference.

After decalcification, histological manifestations of peri-implant bone were assessed. **Figure 4** shows that diminished BIC (F=255.07, df 5 and 24, p<0.01) and B.Ar/T.Ar (F=19.59, df 5 and 24, p<0.01) were observed in the OVX group compared with the NC group, whereas a similar fibrous pseudomembrane formation was found in the two groups (F=60.34, df 5 and 24, p<0.01). Consistent with micro-CT results above, H&E staining and Masson staining revealed that less and thinner bone mass was exhibited around the implant in the O+T group, with abundant fibrous pseudomembrane formation and reduced bone-implant contact when compared with other groups (**Figure 4**). Restored trabecular bone and enhanced interface contact were observed after combination therapy and monotherapy, as well as decreased pseudomembrane appearance. Histomorphological quantification was further performed, confirming the results above. Moreover, pseudomembrane and BIC in the P+Z group were thinner than in the PTH group, while no difference was found between the ZOL and P+Z groups. In addition, no considerable differences were observed in B.Ar/T.Ar among these three groups (**Figure 4C**).

### Effects of Combined Therapy and Monotherapy on Bone Formation and Bone Resorption

**Figure 5:**
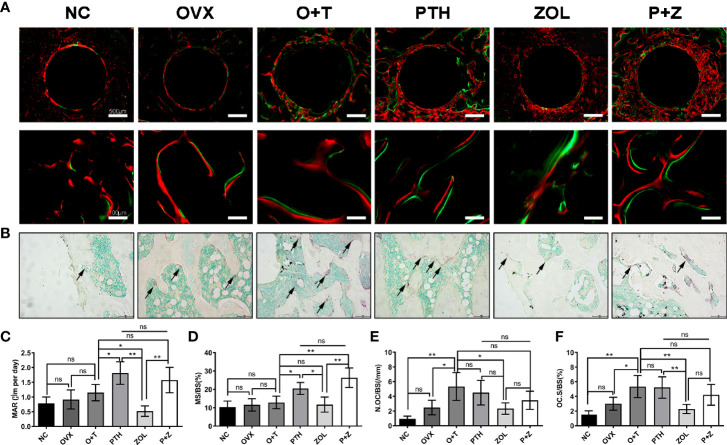
Different effects of combined therapy or monotherapy on bone formation and osteoclast formation in the animal model. **(A)** Representative images of alizarin red (red) and calcein (green) labels were observed. **(B)** Representative images of TRAP staining were presented. The **(C)** MAR, **(D)** MS/BS, **(E)** N.Oc/BS and **(F)** OcS/BS were analyzed with sections. Values expressed are means ± SD, n=5; *p<0.05, **p<0.01, significantly different compared with the O+T group. "ns", no significant difference.

**Figure 6:**
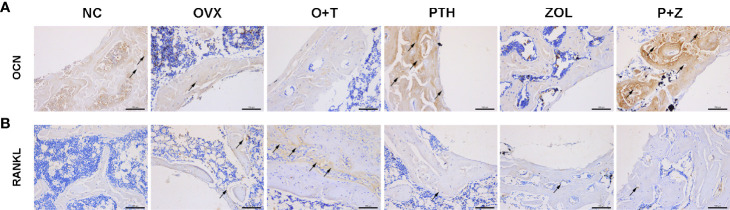
The effects of combined therapy or monotherapy on the expression of OCN **(A)** and RANKL **(B)** in peri-implant bone. Representative IHC staining images of OCN and RANKL were visualized.

**Figure 7:**
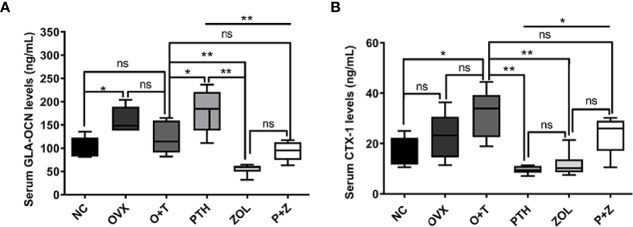
The effects of combined or single treatment on serum levels of GLA-OCN and CTX-1 in the animal model. The serum levels of GLA-OCN **(A)** and CTX-1 **(B)** were measured using ELISA. Values expressed are means ± SD; n=5~6; *p<0.05, **p<0.01, significantly different compared between two groups. "ns", no significant difference.

To investigate the effects of different treatments on bone formation, calcein green and alizarin red were injected successively before euthanasia. As a classical marker for bone formation, double fluorescence labeling was visualized using undecalcified bone section, and MAR (F=12.19, df 5 and 24, p<0.01) and MS/BS (F=13.85, df 5 and 24, p<0.01) were quantified in all groups. As shown in **Figure 5A**, fewer labels were found in the NC group, OVX group, and O+T group than the other groups, whereas Ti particles with drug stimulation enhanced labels. Quantification data indicated no obvious differences in mineral apposition found among the NC, OVX, and O+T groups (**Figures 5C, D**). However, elevated MAR and MS/BS were observed in the PTH and P+Z groups compared to the O+T group, while declined MAR was found in the ZOL group. The immunochemistry staining of OCN further indicated that peri-implant osteoblast activities, inhibited by Ti particles, were reversed by the PTH treatment, with or without ZOL (**Figure 6**). Furthermore, a serum bone formation marker, GLA-OCN, was measured to evaluate the systemic effects of combined therapy and monotherapy (30). **Figure 7A** revealed that, compared with the O+T group, increased serum GLA-OCN levels (F=13.79, df 5 and 27, p<0.01) were found under PTH alone treatment, while ZOL monotherapy decreased the level, consistent with the fluorescence labeling results. Interestingly, no differences were observed between the O+T group and the P+Z group, unlike the mineral apposition data.

TRAP staining was used to examine the influence on periprosthetic osteoclast formation under different treatments. As presented in **Figures 5B**, **E, F**, no difference but an increasing trend of osteoclasts was found in the OVX group in comparison with the NC group. Ti particles significantly stimulated osteoclast formation in the OVX rats, resulting in the elevation of N.OC/BS (F=7.971, df 5 and 24, p<0.01) and OC.S/BS (F=9.520, df 5 and 24, p<0.01). However, a considerable inhibition on the osteoclast number and size was observed with the treatment of ZOL monotherapy, while no differences were demonstrated with combined therapy or PTH monotherapy. Due to the critical role RANKL plays during osteoclastogenesis, we investigated the expression of RANKL around the implants (31). An increased expression of RANKL was found in the peri-implant region in the O+T group, compared with that in the other groups, while combined or single treatment of PTH and ZOL reduced the expression (**Figure 6**). Moreover, serum CTX-1 levels (F=8.667, df 5 and 28, p<0.01) were measured to confirm this further. Unexpectedly, as shown in **Figure 7B**, Ti particle-induced serum CTX-1 level elevation was reversed by both single PTH and single ZOL treatment, whereas no changes were observed in the P+Z group.

## Discussion

In the present study, the combined treatment and monotherapy of PTH and ZOL enhanced periprosthetic bone volume and bone-implant contact and intramedullary implant stability in a debris wear-induced periprosthetic osteolysis under a condition of osteoporosis. Moreover, combined PTH and ZOL therapy revealed an additive effect on preventing periprosthetic osteolysis and improving prosthetic anchorage, exhibiting a greater improvement than monotherapy, even similar or higher than the NC group. Thus, our findings indicated that combination or monotherapy with PTH and/or ZOL might be a promising strategy for preventing early-stage implant loosening in patients with severe osteoporosis.

Various strategies have been attempted to attenuate periprosthetic osteolysis and subsequently implant loosening, including prosthesis modification and systemic agent administration (13). Particularly, agents targeting at regulating bone metabolism have been found effective on implant loosening prevention in animal models, such as sclerostin antibody, alendronate (14, 29). Our group has previously proved the protective effect of intermittent PTH administration or weekly ZOL injection on periprosthetic osteolysis, indicating that these were potential ways to prevent implant loosening (17, 19, 32). Additionally, clinical case reports and trials also provided evidence that agents (such as denosumab and PTH) inhibited periprosthetic bone loss and improved early-stage implant loosening after arthroplasty (20, 21, 33). However, considerable concern should be taken into account, as all these agents are only approved for osteoporosis treatment rather than the treatment of implant loosening. Osteoporosis has been found to increase the morbidity of implant loosening and accelerate the disease progression due to the poor periprosthetic bone quality (8, 9). In this study, we performed the OVX surgery 12 weeks before establishing the debris wear-induced periprosthetic osteolytic model. Female SD rats weighing 200–250 g are regarded as sexually mature preoperatively, in which bone remodeling prevails to bone modeling and is suitable for OVX surgery (34). Simultaneously, constant bone mass was reported to be observed in rats 12 weeks after OVX previously, with a significant bone loss, indicating that the animal model was approaching to aged- or postmenopausalrelated osteoporosis clinically (35). Reduced bone mass, atrophic uterus, and elevated weight observed 12 weeks after OVX surgery in our study certified the successful establishment of the animal model of osteoporosis, meeting the indications of anti-osteoporotic drugs. The subsequent surgery for implant loosening was performed based on the osteoporotic model afterward. A clinical study has shown that osteoporosis is found to be a comorbidity with aseptic loosening (9). Consistent with this clinical study, our research demonstrated that significantly decreased maximal pull-out force and peri-implant bone mass were observed in the OVX group, further confirming that osteoporosis might increase the chance of aseptic loosening. Moreover, a murine study proved that osteoporosis aggravated Ti particle-induced calvarial osteolysis *in vivo* (18). In our research, increased pseudomembrane and decreased BIC were observed between the OVX group and the O+T group, while no significant differences but downregulated trends were found in biomechanical strength and periprosthetic bone mass. The dense pseudomembrane formation and reduced bone contact confirmed that the periprosthetic osteolytic model has also been built successfully. By contrast, biomechanical strength and periprosthetic bone mass results exhibited no differences but reduced trends, which might be caused by 12-week OVX-induced severe bone loss. Although debris wears accelerated bone loss, it might attenuate the variation between the OVX groups with and without Ti particles.

ZOL, a representative bisphosphonate, serves as the first-line antiosteoporotic agent clinically worldwide. Several nationwide clinical studies clarified that bisphosphonate in patients with THA exhibited a lower risk for revision surgery, indicating that bisphosphonate use is encouraged in patients suffering from osteoporosis and candidates for THA (25, 36). Animal studies also proved that both local and system administrations of bisphosphonate displayed a capacity to prevent particle-induced osteolysis by diminishing bone loss and fiber formation (18, 19, 32). In this study, the systemic administration of ZOL has not improved implant anchorage compared with the O+T group. However, micro-CT and histological results demonstrated that periprosthetic bone loss induced by the synergistic effect of osteoporosis and particles was reversed markedly under ZOL treatment *via* manifesting the increased BMD, BV/TV, Tb.N, Tb.Th, and Conn.D, as well as reduced BS/BV and Tb.Sp. Inflammation and osteoclastic resorption play crucial roles in osteoporosis and particle-induced osteolysis, resulting in excessive bone destruction. ZOL has confirmed the suppressive effects on bone loss in several animal models, aiming to inhibit osteoclast differentiation and osteoclastic function and induce apoptosis by modulating the mevalonate pathway and diminishing adherence to osteoclast onto the bone surface (37, 38). According to those studies above, in our research, the obviously elevated number and size of osteoclasts were confirmed under the synergistic action of osteoporosis and periprosthetic osteolysis while they were impaired after ZOL treatment. Mechanically, RANKL initiates the activation of signaling cascades and drives osteoclast differentiation and function (39). Ramage et al. indicated that RANKL was highly expressed in fibroblast cells located at the periprosthetic membrane, regulating focalized bone resorption (40). Our IHC results showed the abundant expression of RANKL in the pseudomembrane, whereas ZOL administration attenuated RANKL expression in fibrous tissues. In addition, reduced fibrous pseudomembrane formation was observed under ZOL administration. Since bisphosphonates have not exhibited inhibitory effects on wear debris-induced inflammation in a dog model and human specimens, the explanation was that ZOL decreased osteoclastic resorptive regions, which was presumably filled with fibrous tissues formed by ongoing inflammation-facilitated cell necrosis and fibrosis (41, 42). The inhibition of bone formation by ZOL has been reported previously that ZOL downregulates the proliferation, differentiation, maturation, and function of osteoblasts (23, 43). Our findings further demonstrated a similar result that ZOL reduced the bone formation rate and OCN expression in this animal model. Simultaneously, the serum level of GLA-OCN and CTX-1 further confirmed the suppressive effects of ZOL on both bone formation and bone resorption. Thus, our result demonstrated that ZOL could inhibit peri-implant bone loss but played a limited role in preventing implant loosening.

A considerable enhancement on implant fixation was exhibited under daily PTH treatment, compared with that in the O+T group, with improved stiffness and maximal pull-out force. Several studies from our group and other groups have exhibited beneficial effects on improving prosthetic fixation (17, 32, 44). Recently, some case reports also indicated the clinical efficacy of PTH on the improvement of early-stage implant loosening, with the disappearance of the radiographic line, which is consistent with our findings (20–22). Moreover, PTH showed a more substantial beneficial effect on the biomechanical test than ZOL in this study, which has been proven in other studies before (45). On the contrary, the local administration of ZOL contributed a better potential than PTH on the maximal push-out force in a rat model of initial stability, suggesting that different methods of administration might produce different efficiencies (44). Increased periprosthetic bone mass and decreased pseudomembrane formation are considered to contribute to the enhancement of implant stability (26). PTH is a commonly used anabolic agent on bone remodeling regulation, demonstrating accelerative effects on osteoblastic bone formation and osteoclastic resorption (46). Furthermore, intermittent administration of PTH has shown a greater promotive effect on bone formation than bone resorption, resulting in an elevation of bone mass (47). In this study, elevated trabecular bone and reduced periprosthetic fibrous membrane formed around the implants after PTH treatment, with a significantly raised bone formation rate. The presence of wear debris stimulates the process of chronic inflammation, which disrupts bone formation by impairing osteoblastic proliferation, differentiation, and maturation, as well as inducing apoptosis (48). PTH has been reported to enhance cell activity, prolong the lifespan, reduce the apoptosis of osteoblasts, and promote osteogenic differentiation of MSCs, which greatly supports our findings (49, 50). A higher expression of OCN in peri-implant bone and serum with PTH treatment further confirmed the anabolic effects on bone formation. In addition, less fibrous tissue was observed in the BIC region under PTH treatment, providing better contact between bone and the implant. This finding was consistent with the results reported in a previous study, in which the authors found that PTH exerted a suppressive effect on fibrosis (51).

The exact effects of combined administration with PTH and ZOL remain controversial. No additive effect of PTH and alendronate was exerted in the treatment of osteoporosis in men or postmenopausal women (52, 53); However, Cosman et al. found that the combination therapy of PTH (20 μg daily) and ZOL (5 mg per year) showed an additive effect and a substantial increments in the BMS of spine and hip (54). In addition, some animal studies also confirmed the beneficial effects of combination therapy on promoting implant fixation, preventing disuse-induced osteopenia, and improving posterior lumbar vertebral fusion, compared with PTH or ZOL monotherapy (23, 24, 44). Thus, we hypothesized that conjunctive use of PTH and ZOL exhibited a better preventive effect than PTH or ZOL alone on particle-induced implant loosening in a rat model with osteoporosis. In the present study, the combined PTH with ZOL was found to be effective in maintaining peri-implant bone mass and facilitating the stability of the implant, which is in accordance with the previous studies. Outstanding improvement of maximal pull-out force and stiffness was observed in the combination therapy group, with a similar efficiency with PTH alone but higher than in the ZOL and NC groups. The additive effect on bone mass raise was also exhibited in micro-CT and histomorphological results, along with significantly decreased pseudomembrane thickness. Nevertheless, the major concern is whether the anabolic effect of PTH would be blunted when combined with bisphosphonates (55). Our study demonstrated a similar anabolic effect with the PTH group, which is not impaired obviously by ZOL administration. On the other hand, ZOL-induced inhibition of osteoclasts was slightly counteracted by the utilization of PTH, which is confirmed by the serum results and TRAP staining, resulting in good bone homeostasis. Unexpectedly, the inhibited serum CTX-1 level by PTH or ZOL was reversed with the combination therapy at the level of the NC group. There is no plausible explanation for the interesting phenomenon. Taken together, the combination therapy of PTH and ZOL exerted a superior effect than monotherapy, with better fixation strength and peri-implant bone mass and restoring the bone turnover to the normal level.

Various cells are involved in the process of periprosthetic osteolysis, such as osteoclasts, osteoblasts, fibroblasts, and osteocytes. Osteoblasts and osteoclasts play crucial roles in regulating bone metabolism, and reactions were observed in osteoblasts and osteoclasts after particle wear stimulation during the process of prosthetic osteolysis. Chemokines and proinflammatory cytokines, including tumor necrosis factor (TNF)-α, interleukin (IL)-1β, IL-6, IL-11, and macrophage colony-stimulating factor (M-CSF), were secreted in the presence of debris wears, which is in response to an innate host immune stimulation, resulting in excessive osteoclast formation and activity (56, 57). RANKL-RANK-osteoprotegerin (OPG) axis plays an essential role in osteoclastogenesis, while TNF-α and IL-1β support the survival, differentiation, and activation of osteoclasts (58, 59). When RANKL binds to RANK, a series of downstream signaling cascades are initiated, including the ERK, p38, JNK, and NF-κB pathways, subsequently promoting the auto-amplification of the nuclear factor of activated T cells, cytoplasmic, calcineurin-dependent 1 (NFATc1, the key molecule of osteoclastogenesis), resulting in osteoclastogenesis (26). ZOL has been widely used to treat osteoporosis with preventive effects on osteoclastogenesis. Inhibiting farnesyl diphosphate (FPP) synthase, a key enzyme of the mevalonate pathway, is generally considered the main molecular mechanism of ZOL on suppressing osteoclast formation antiresorptive potency (60). With a similar mechanism of osteoclastogenesis in periprosthetic osteolysis and osteoporosis, the suppression of the mevalonate pathway might also be the mechanism of ZOL on preventing periprosthetic osteolysis. In the meantime, osteoblast functions are impaired under particle wear stimulation, exhibiting supranuclear vacuolization, cell cycle arrest, and elevated DNA damage (61). Moreover, the adverse effects of viability, proliferation, adhesion, migration, osteogenic differentiation, and mineralization on osteoblasts were also observed in the presence of particle wear (48). In the mechanism, wnt/β-catenin and BMP/Smad signaling pathways were impaired by particles during osteogenic differentiation (62). In addition, OPG, secreted by osteoblasts, is found to be suppressed in periprosthetic osteolysis, resulting in the imbalance of OPG/RANK/RANKL axis (63). Teriparatide (PTH 1-34) has exhibited its capacity to improve bone formation *via* PKA (protein kinase A) and Wnt/b-catenin pathways and also stimulate OPG secretion (64). Here, we think that the Wnt/β-catenin pathways and OPG/RANK/RANKL axis might be involved in the mechanism of PTH-treated periprosthetic osteolysis by improving osteogenic differentiation, viability, proliferation, adhesion, and migration, as well as attenuating DNA damage.

Some limitations are worth being concerned to our study. Although polyethylene wear particles are considered the leading cause for implant loosening rather than metal particles, metal particles still play a role. They are confirmed to be effective during the initiation and process of peri-implant osteolysis *in vivo* and *in vitro* (65). Thus, we used Ti particles here to establish the animal model. In addition, the 6-week duration of particle-induced osteolysis could not represent the entire process of pathological changes. A study with a long period is needed further. Moreover, as the maximum treatment duration with PTH is approved to 24 months in lifespan and the withdrawal of PTH has been proven to lead to the deterioration of implant fixation in a particle-induced osteolytic model, it would be valuable to explore whether the usage of ZOL should be continued when withdrawing PTH, after a period of combined PTH with ZOL (32, 66).

In conclusion, Ti particles deteriorated implant fixation strength, along with periprosthetic bone loss and increased pseudomembrane formation in an OVX rat model. PTH monotherapy or combined PTH with ZOL exerted a protective effect on maintaining implant stability by elevating periprosthetic bone mass and inhibiting pseudomembrane formation *via* regulating bone metabolism. Moreover, an additive effect was observed when combining PTH with ZOL, resulting in better fixation strength, higher periprosthetic bone mass, and less pseudomembrane than PTH monotherapy. Taken together, our results suggested that combination therapy of PTH and ZOL might be a promising approach for the intervention of early-stage implant loosening in patients with osteoporosis.

## Data Availability Statement

The original contributions presented in the study are included in the article/**Supplementary Material**. Further inquiries can be directed to the corresponding authors.

## Ethics Statement

The animal study was reviewed and approved by the Institutional Animal Care and Use Committee of the Second Affiliated Hospital of Zhejiang University School of Medicine.

## Author Contributions

CZ, SY, HW, and MS: designed the experiment. CZ, YW, JM, FB and HZ: performed the experiment. MY and HX: performed the measurement and analysis. CZ, GY, and CW: drafted the manuscript. CZ and MS: revised the manuscript. All authors contributed to the article and approved the submitted version.

## Funding

This study was supported by research grants from Zhejiang Natural Science Foundation (No.LQ21H060006), the National Natural Science Foundation of China (No.82001461, No.81902279, No.82102628), and the fellowship of China Postdoctoral Science Foundation (No.2020M671758, No. 2021M692795).

## Conflict of Interest

The authors declare that the research was conducted in the absence of any commercial or financial relationships that could be construed as a potential conflict of interest.

## Publisher’s Note

All claims expressed in this article are solely those of the authors and do not necessarily represent those of their affiliated organizations, or those of the publisher, the editors and the reviewers. Any product that may be evaluated in this article, or claim that may be made by its manufacturer, is not guaranteed or endorsed by the publisher.
