# Supplementary material for: Additive Effect of Parathyroid Hormone and Zoledronate Acid on Prevention Particle Wears-Induced Implant Loosening by Promoting Periprosthetic Bone Architecture and Strength in an Ovariectomized Rat Model
Source: Front Endocrinol (Lausanne). 2022 Apr 25;13:871380. doi: 10.3389/fendo.2022.871380 (PMC9084285; doi:10.3389/fendo.2022.871380)
Supplement: Supplementary file 1 [file Image_1.pdf]

Supplementary Material

Additive effect of parathyroid hormone and zoledronate acid on prevention particle wears-induced implant loosening by promoting periprosthetic bone architecture and strength in an ovariectomized rat model

Chenhe Zhou[1,2,3*], Yangxin Wang[1,2,3*], Jiahong Meng[1,2,3], Minjun Yao[1,2,3], Huikang Xu[4], Cong Wang[1,2,3], Fanggang Bi[5], Hanxiao Zhu[1,2,3], Guang Yang[1,2,3], Mingmin Shi[1,2,3#], Haobo Wu[1,2,3#], Shigui Yan[1,2,3#]

1. Department of Orthopedic Surgery, The Second Affiliated Hospital, Zhejiang University School of Medicine, Hangzhou, China.

2. Orthopedic Research Institute of Zhejiang University, Hangzhou, China.

3. Key Laboratory of Motor System Disease Research and Precision Therapy of Zhejiang Province, The Second Affiliated Hospital, Zhejiang University, Hangzhou, China.

4. State Key Laboratory for Diagnosis and Treatment of Infectious Diseases, National Clinical Research Center for Infectious Diseases, Collaborative Innovation Center for Diagnosis and Treatment of Infectious Diseases, The First Affiliated Hospital, College of Medicine, Zhejiang University, Hangzhou, China.

5. Department of Orthopaedic Surgery, The First Affiliated Hospital of Zhengzhou University, Zhengzhou, China

Supplementary Figure 1

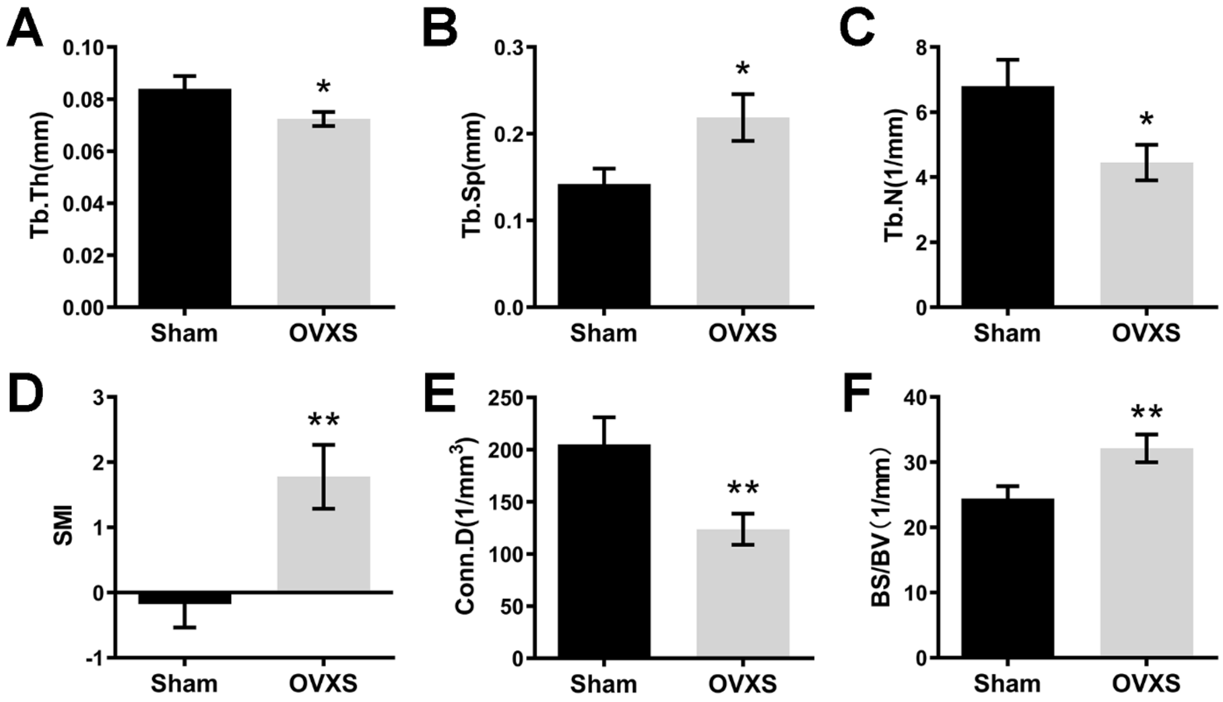

Supplementary Figure 1. The micro-CT results of distal femurs in the Sham and OVXS groups. (A-F) The quantification of the BS/BV, Conn.D, SMI, Tb.N, Tb.Th and Tb.Sp values were analyzed. Values expressed are means ± SD, n=5; $*p < 0.05$, $**p < 0.01$, significantly different compared between two groups.
